# Imaging carbonic anhydrase IX as a method for monitoring hypoxia-related radioresistance in preclinical head and neck cancer models

**DOI:** 10.1016/j.phro.2021.08.004

**Published:** 2021-08-24

**Authors:** Fokko J. Huizing, Bianca A.W. Hoeben, Jasper Lok, Otto C. Boerman, Sandra Heskamp, Johan Bussink

**Affiliations:** a Department of Radiation Oncology, Radboud University Medical Center, Nijmegen, The Netherlands; b Department of Radiology and Nuclear Medicine, Radboud University Medical Center, Nijmegen, The Netherlands

**Keywords:** Head and neck xenografts, Hypoxia, CAIX imaging, Functional imaging, Girentuximab, Atovaquone

## Abstract

**Background and purpose:**

Tumor hypoxia is an important cause of radioresistance and is associated with poor outcome.

SPECT (Single-photon emission computed tomography) imaging enables visualizing tumor characteristics. We investigated the SPECT-radiotracer [^111^In]-girentuximab-F(ab’)_2_ to image Carbonic Anhydrase IX (CAIX), an enzyme upregulated under hypoxic conditions.

**Materials and methods:**

Athymic mice with subcutaneous FaDu or SCCNij202 head and neck squamous cell carcinoma (HNSCC) xenografts were treated with atovaquone or were housed in a hypoxic chamber (8% O_2_). Next, [^111^In]-girentuximab-F(ab’)_2_ was injected and 24 h later mice were euthanized for *ex vivo* biodistribution, autoradiography of the tumor, and immunohistochemical staining of the tumor. Tumor sections were analyzed for hypoxia, CAIX expression, vessels, and perfusion. Also, the effect of atovaquone on microSPECT scans was determined in the FaDu model.

**Results:**

Atovaquone decreased CAIX expression by 69% (p = 0.017) compared with control tumors in FaDu, while in the SCCNij202 tumors no difference was observed. Hypoxic breathing did not increase CAIX expression or hypoxia staining in either tumor model, but did affect the necrotic tumor fraction. *Ex vivo* tracer uptake in the atovaquone treated group did not differ significantly from the control group, despite the difference in CAIX expression. Furthermore, SPECT imaging with [^111^In]-girentuximab-F(ab’)_2_ did not discriminate atovaquone-treated versus control tumors.

**Conclusion:**

Atovaquone decreased CAIX expression only in the FaDu tumor model. [^111^In]-girentuximab-F(ab’)_2_ specifically targets CAIX-expressing areas in HNSCC xenografts, but differences in vessel density and necrosis most likely affected tracer uptake in the tumors and therefore complicated quantification of changes in CAIX expression.

## 1 Introduction

Locally advanced head and neck squamous cell carcinomas (HNSCC) are treated by surgery and/or radiotherapy, often combined with concurrent chemotherapy. Unfortunately, therapy response is inconsistent, resulting in a poor 5-year overall survival of approximately 50% [1]. Better predicting and monitoring of therapy response may allow early and personalized modification of the treatment plan.

A prominent predictor of therapy resistance for non-surgical treatments is tumor hypoxia. Both chemotherapy and radiotherapy are less effective in tumors with inadequate oxygenation [2], [3]. Therefore, the hypoxic status of tumors and the development of new anti-hypoxia treatments have been subjects of investigation over the past decades.

Multiple therapies have been evaluated for their potential to modulate tumor oxygenation. Breathing of oxygen-enhanced gas mixtures (e.g. carbogen) [4], hypoxia mimicking agents (nimorazole) [5], hypoxia activated pro-drugs (tirapazamine and TH-302) [6], [7], and drugs inhibiting oxygen consumption (metformin and atovaquone [17]) have been shown to have an effect on tumor hypoxia. Nevertheless, none of these treatments became part of clinical practice yet - except for nimorazole in Denmark - due to non-conclusive clinical studies, albeit that meta-analyses have shown the clinical benefit of hypoxia modifying treatments in combination with radiotherapy [5], [8].

In the path towards more individualized hypoxia-targeted therapies, predictive biomarkers will likely play a substantial role for better selection of such patients. Assessment with molecular imaging has the ability to visualize biochemical processes in entire primary tumors and metastases. This is crucial for the assessment of hypoxia since it is a heterogenous phenomenon between and within tumors. It is heterogeneously present within tumors and can change over time due to disease progression and therapy. Molecular imaging has the potential to repeatedly assess tumor hypoxia and thereby allows making early therapy adjustments. For example, if the hypoxic status of a tumor does not improve, the selected modification can be adapted.

PET (positron emission tomography) and SPECT (Single-photon emission computed tomography) imaging enables us to visualize tumor characteristics. Currently, PET is the most commonly used technique to assess tumor hypoxia, making use of 2-nitromidazol-based radiotracers (FMISO, FAZA and HX4) [9], [10]. Their accumulation mechanism depends on their reductive metabolism, which occurs irreversibly only under hypoxic conditions, and thereby entraps the tracer in the hypoxic tumor regions. This imaging method has its limitations due to low contrast between hypoxic and non-hypoxic subvolumes and its variable reproducibility [11]. This lack of reproducibility might be explained by the fact that tumor hypoxia is a dynamic phenomenon in time and place. Differences in perfusion limited hypoxia may result in differences in uptake patterns of 2-nitromidazol-based radiotracers over time.

A different approach is to image relevant viable radioresistant cells in hypoxic tumor areas, by targeting a hypoxia-related antigen expressed by tumor cells adapted to hypoxic conditions. A candidate antigen for this approach is Carbonic anhydrase IX (CAIX). It is a downstream target of the hypoxia inducible factor alpha (HIF1-α) and is expressed on the cell membrane in a time frame of several hours after onset of hypoxia. Therefore, it represents a subpopulation of cells which have adapted to a hypoxic environment. CAIX has been proven to be a prognostic biomarker in almost all solid malignancies [12].

Previously, our group developed a quantitative CAIX imaging technique using the SPECT tracer [^111^In]In-G250-F(ab’)_2_ [13]. We demonstrated that this technique can discriminate low CAIX-expressing tumors from high CAIX-expressing tumors [14]. Therefore, it could play a predictive role for treatments where hypoxia is involved in therapy resistance. However, it remains unclear if this radiotracer is also capable of detecting changes in the level of CAIX expression within the same tumor model. To answer this question, we set up experiments where we modulated the level of oxygenation towards an improved oxygenation status (atovaquone) and a decreased oxygenation status (hypoxic gas breathing) in two different human HNSCC models. We evaluated whether this change in oxygenation status altered the CAIX expression levels and tumor targeting of [^111^In]In-G250-F(ab’)_2._

## 2 Materials and methods

### 2.1 Radiotracer production

[^111^In]In-G250-F(ab’)_2_ was produced and radiolabeled as described previously [15]. In short, cG250 was enzymatically digested with the use of pepsine, resulting in F(ab’)_2_ antibody fragments. The (Fab’)_2_ fragments (110 kDa) were purified and subsequently conjugated with diethylenetriamine pentaacetate (DTPA). At days of tracer injection, DTPA-G250-F(ab’)_2_ was radiolabeled with In-111 (Curium, Petten, The Netherlands) at an average specific activity of 1.1 MBq/ug. The radiochemical purity exceeded 95% in all experiments.

### 2.2 Animal models

Female athymic BALB/c nude mice mice (Janvier Labs, Le Genest-Saint-Ile, France) were aged six to eight weeks and weighed 18–22 g at the start of the experiments. The animals were housed together in filter-topped cages in a specific pathogen-free unit and were included in the experiments when tumors had a mean diameter of 5 mm. Stratified randomization was used based on tumor size, in order to have a similar mean tumor size per study group. The studies were approved by the Central Authority for Scientific Procedures on Animals (RU-DEC-2016-053) and carried out under supervision of the local Animal Welfare Body.

### 2.3 Tumor inoculation

Two HNSCC tumor models were used. The SCCNij202 model is a patient-derived HNSCC xenograft model derived from a patient with larynx carcinoma. We have previously demonstrated that this is a hypoxic tumor model with high CAIX expression [15]. Mice were implanted on the right hind leg with a small (2 mm diameter) tumor chunk excised from a donor mouse. The tumors grew to a size of 5–7 mm in circa four to five weeks.

The second tumor model was the FaDu model. This is a well-known HNSCC cell line, and is also known to be hypoxic and capable of expressing moderate to high levels of CAIX. FaDu cells were cultured at 37 °C with 5% CO_2_ in medium (DMEM, Sigma) supplement with 10% fetal bovine serum. Mice were inoculated subcutaneously with 1 million FaDu cells suspended in 50 μl medium and 50 μl Matrigel (BD Biosciences). The tumors grew to a size of 5–7 mm in circa two weeks.

### 2.4 Hypoxia modulating treatment

To modulate tumor oxygenation in tumor-bearing mice, the following interventions were performed: Atovaquone and hypoxic breathing. Three treatment groups of five mice were evaluated in the two different tumor models, resulting in 6 groups in total. Atovaquone suspension (Wellvone) 50 mg/kg, to reduce hypoxia by modulating mitochondrial function, was administered orally with the use of a gavage, daily on 8 consecutive days. Hypoxic breathing setup consisted of an airtight cage with an inlet tube and outlet tube. Via the inlet an 8% oxygen and 92% nitrogen gas mixture flowed at a minimal rate (155 l/h). The outlet tube had an oxygen sensor which displayed the current oxygen percentage on demand. Mice were kept under these conditions for 48 h, and only taken out for the i.v. administration of the radiotracer.

### 2.5 Biodistribution studies

Biodistribution studies were performed as described for our previous studies with [^111^In]In-G250-F(ab’)_2_ [14]. Briefly, all mice were injected i.v. with 10 μg [^111^In]In-G250-F(ab’)_2_ (1 MBq) diluted with PBS 0.5% BSA to a volume of 200 μl, on the second to last day of treatment and 24 h before euthanasia. Mice were euthanized by CO_2_/O_2_ inhalation and tumors and normal tissues were collected. Half of the tumor was used for biodistribution analysis, while the other half was used for immunohistochemistry and autoradiography. Samples were weighed and subsequently the radioactivity in the tissue samples was determined in a γ-counter (2480 Wizard 3″, LKB/Wallace, Perkin-Elmer, Boston, MA). Activity concentrations in the tissues were calculated as percentage of the injected dose per gram of tissue (%ID/g). Tracer injection standards were measured simultaneously for decay correction.

### 2.6 MicroSPECT imaging

In a separate experiment 10 other animals were used for the acquired SPECT scans. These mice, bearing a FaDu tumor, were allocated to one of two groups (n = 5 each); a control group and an atovaquone treatment group. The procedure was similar to the first experiment, except mice were scanned with microSPECT at 23 h after injection with 10 μg [^111^In]In-G250-F(ab’)_2_ (10 MBq). Images were acquired with microSPECT (U SPECT-II; MILabs, Utrecht, The Netherlands). Animals were anesthetized with isoflurane and the tumor region (to maximize signal) was scanned in prone position using a 1.0 mm diameter multipinhole mouse collimator. Scans were acquired for 50 min using twelve bed positions. Scans were reconstructed using MILabs reconstruction software with five iterations, sixteen subsets and an algorithm with a voxel size of 0.375 mm. Subsequently, SPECT images were analyzed with Inveon Research Workplace software (version 3.0; Siemens Preclinical Solutions).

### 2.7 Immunohistochemistry and autoradiography

Half of the excised tumors were immediately snap-frozen in liquid nitrogen and cut into 5 µm sections. Subsequently, tissue sections were mounted on poly-l-lysine coated slides. Sections were fixed with cold acetone for 10 min at 4 °C. For every tumor, marked sections were analyzed by autoradiography and immunohistochemical staining. Overnight, sections were exposed to a phospholuminescence plate in a Fujifilm BAS cassette 2025 (Fuji Photo Film), after which the plates were scanned with a BAS-1900 II bioimaging analyzer and analyzed with Aida Image Analyzer software (Raytest). Then, slides were stored at −80 °C. Immunohistochemical staining for CAIX, hypoxia (pimonidazole), vessels and perfusion (Hoechst 33342) was performed as described previously Hoeben et al. [16]. Staining for hypoxia and CAIX was quantified in fraction of the viable tumor area multiplied by the signal intensity, thereby taking in to account both the number of CAIX positive cells and the intensity of the staining. Necrotic fractions were determined as fraction of the total tumor area with the help of H&E staining of consecutive tumor sections [16].

### 2.8 Statistics

Graphpad Prism 6 was used for statistical analyses. Data were presented as mean and standard deviation. Statistical significance was determined with the one-way ANOVA. The Dunnett's correction was used in case of multiple testing. Correlation was assessed by using the Pearson test. A p-value of <0.05 was used to determine significance.

## 3 Results

### 3.1 Modulation of hypoxia and CAIX by atovaquone and low oxygen gas breathing

**Fig. 1:**
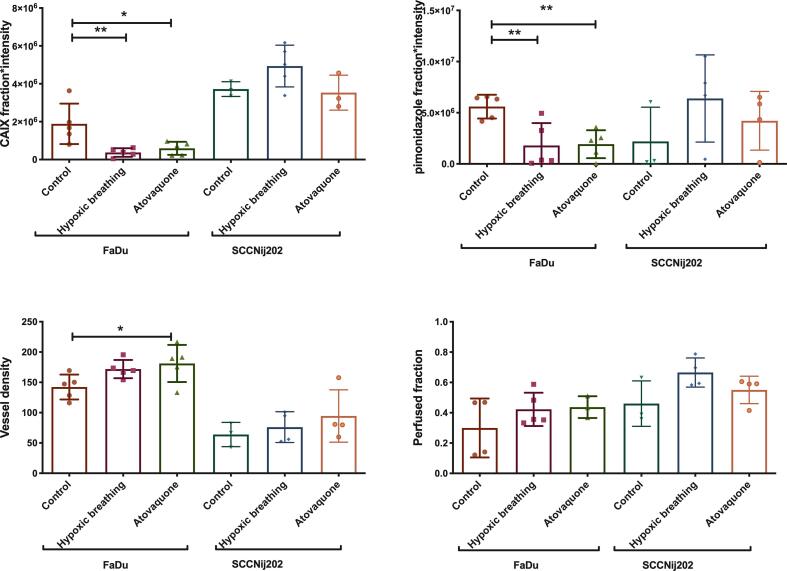
Immunohistochemical analysis of the tumors in the 3 treatment groups in both the FaDu and SCCNij202 tumor models. CAIX staining and pimonidazole staining are expressed in fraction of the viable tumor × mean signal intensity. (A) CAIX expression, (B) pimonidazole, (C) Vessel density (vessels per mm^2^) and (D) Perfused fraction. Graph bars represent mean ± SD (n = 3–5).

Immunohistochemical analyses showed a significant lower staining for CAIX (p = 0.017) and pimonidazole staining (p = 0.007) in atovaquone-treated FaDu tumors compared with non-treated FaDu tumors. The control group had a CAIX staining (fraction × mean intensity) of 1.89 × 10^6^ versus 5.94 × 10^5^ for the treated group: a reduction of 69%. Pimonidazole staining (fraction × mean intensity) was 5.60 × 10^6^ in the control group and 1.93 × 10^5^ for the group treated with atovaquone, a reduction of 66%. Immunohistochemical analyses of the SCCNij202 tumors did not show a significant difference in pimonidazole and CAIX staining of treated and non-treated tumors (Fig. 1A and B). Atovaquone treatment did not have a significant effect on the Hoechst perfused fraction of the tumor in both models, but we did find a significant increase in vessel density in the FaDu model (p = 0.04) (Fig. 1C and D).

**Fig. 2:**
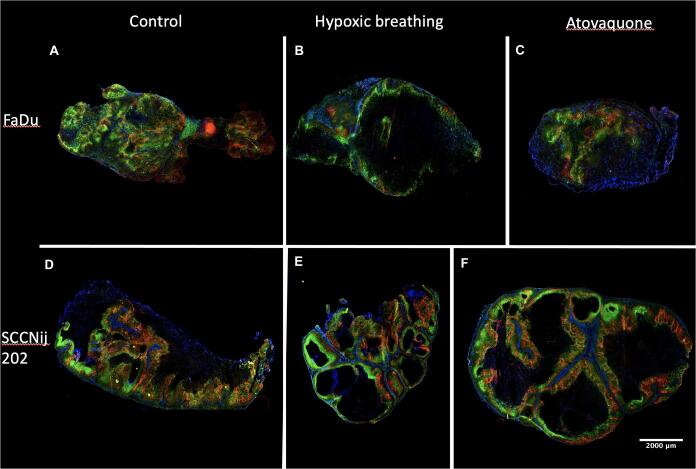
Immunohistochemistry images of all tested conditions. Stained tumor sections of the FaDu model are depicted on the top row as (A) control, (B) after 48 h of 8% oxygen breathing, and (C) after 8 days of atovaquone treatment. On the bottom row, tumor sections of the SCCNij202 model with treatment groups are depicted in the same order. Tumor sections were stained for CAIX in red, pimonidazole in green and vessels in blue. (For interpretation of the references to colour in this figure legend, the reader is referred to the web version of this article.)

**Fig. 3:**
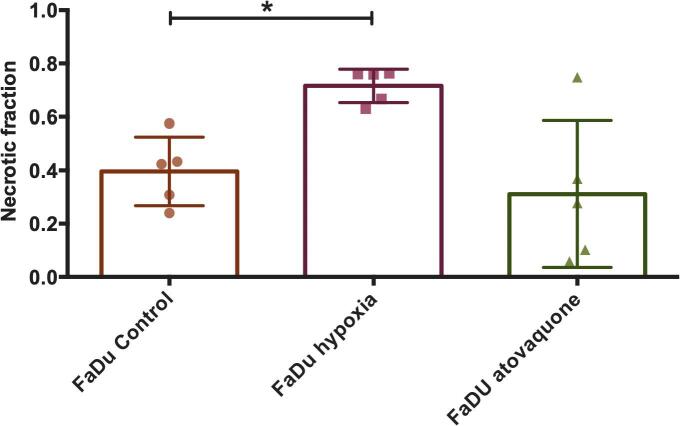
Necrotic fractions of the FaDu tumor model of all 3 treatment groups: control, hypoxic breathing and atovaquone, calculated as fraction of the complete tumor area. Graph bars represent mean ± SD (n = 5).

Hypoxic breathing decreased CAIX staining significantly and was 3.7 × 10^5^ compared with 1.89 × 10^6^ for control FaDu tumors (p < 0.05), a reduction of 70%. Pimonidazole staining also showed a significantly lower signal with 1.80 × 10^6^ compared with 5.60 × 10^6^ (p < 0.05); a reduction of 80% (Fig. 1A and B). Furthermore, FaDu tumors showed a significantly higher necrotic fraction for the hypoxic breathing group compared with the control group (0.40 versus 0.72) (p < 0.01). The necrotic fraction in the atovaquone group (0.31) did not differ significantly from the control group (Figs. 2B and 3).

### 3.2 Tracer uptake in the tumor did not increase after atovaquone treatment

**Fig. 4:**
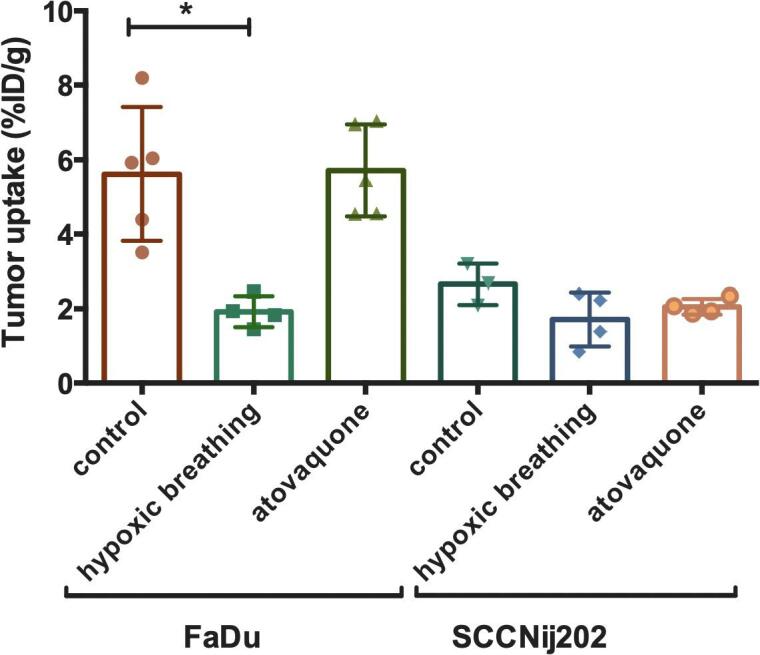
Biodistribution results of tumor tracer uptake 24 h after injection of [^111^In]-girentuximab-F(ab’)_2_ (ID/g) in all 3 treatment groups in both the FaDu and SCCNij202 model. Graph bars represent mean ± SD (n = 3–5).

Tumor tracer uptake in non-treated tumors was 5.61 ± 1.79%ID/g and 2.59 ± 0.47%ID/g for FaDu and SCCNij202, respectively. Atovaquone treatment did not result in a decreased [^111^In]In-G250-F(ab’)_2_ tracer accumulation in either tumor model and resulted in uptake of 5.71 ± 1.23 and 2.05 ± 0.20%ID/g for FaDu and SCCNij202, respectively. In the groups treated with hypoxic breathing, tumor accumulation was significantly lower for the FaDu model with 1.92 ± 0.42%ID/g (p < 0.01) and lower but not significant for the SCCNij202 group with 1.71 ± 0.73%ID/g (p = 0.10) (Fig. 4). Irrespective of treatment, tumor accumulation of [^111^In]In-G250-F(ab’)_2_ (%ID/g) moderately correlated to the level of CAIX staining for all the individual FaDu tumors, with a correlation coefficient of r = 0.49 (p = 0.08) (Sup. Fig. 1).

### 3.3 SPECT imaging did not allow for discrimination of atovaquone-treated versus control tumors

**Fig. 5:**
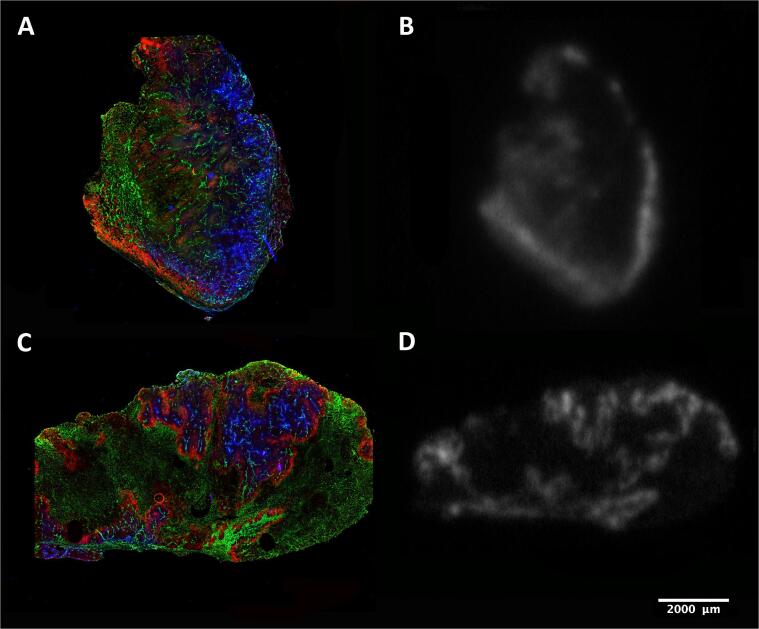
(A) and (C) show a combined immunohistochemistry staining for CAIX (red), tissue perfusion (blue) and vessels (green) as visualized with Hoechst. (B) and (D) are *ex vivo* autoradiography images showing the 2D uptake of [^111^In]-girentuximab-F(ab’)_2_ . (For interpretation of the references to colour in this figure legend, the reader is referred to the web version of this article.)

**Fig. 6:**
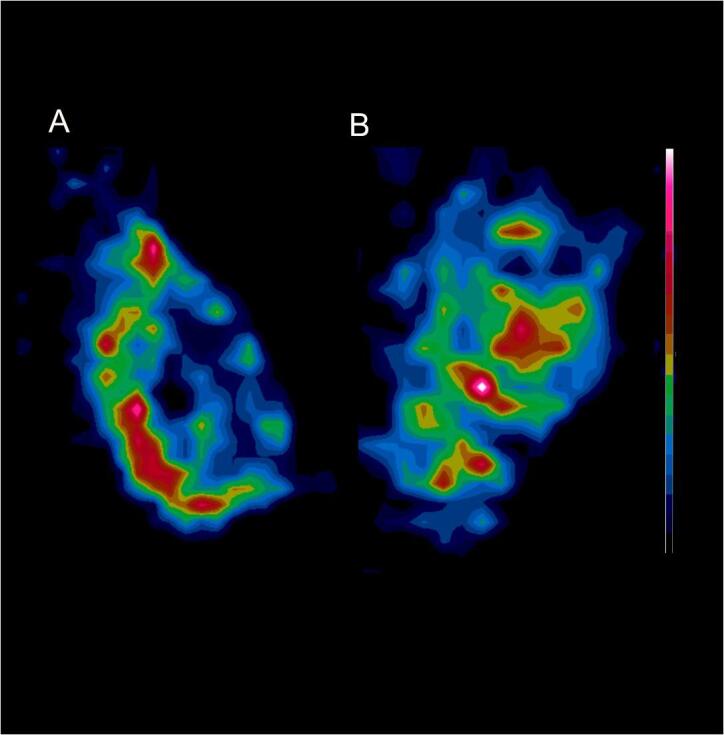
*In vivo* SPECT images show cross sections of the 3D uptake of [^111^In]In-girentuximab-F(ab’)_2_. Image (A) is from a non-treated tumor of the FaDu model and image (B) is from a FaDu tumor treated with atovaquone.

SPECT imaging demonstrated a heterogenous uptake of [^111^In]In-G250-F(ab’)_2_ in the FaDu tumors. The pattern of tracer uptake in the autoradiography images was similar to the CAIX expression pattern as shown immunohistochemically. Apart from this similar pattern, a high signal was observed in non-CAIX-expressing tumor areas as well. No obvious difference in signal intensity was seen between the atovaquone treatment group and the control group (Fig. 5, Fig. 6).

## 4 Discussion

In this study we explored the application of the tracer [^111^In]In-G250-F(ab’)_2_ to assess the changes in CAIX expression induced by hypoxic gas breathing (meant to induce hypoxia and hence increase CAIX expression) and inhibition of mitochondrial respiration with atovaquone to reduce hypoxia (and reduce CAIX expression) in human xenografted HNSCC. We showed that atovaquone treatment reduced tumor hypoxia and CAIX expression in one of the two HNSCC models investigated. However, this reduction could not be detected by CAIX-imaging with [^111^In]In-G250-F(ab’)_2_. Furthermore, we found that atovaquone affected the vessel density and hypoxic breathing affected the amount of necrosis. Our data suggest that changes in vessel density or blood perfusion induced by the treatment may hamper the applicability of [^111^In]In-G250-F(ab’)_2_ for monitoring tumor hypoxia. Lastly, this study demonstrates that, while investigating hypoxia and tumor accumulation of radiotracers, equal pharmacodynamic conditions of test subjects is of vital importance.

In the FaDu model, atovaquone treatment resulted in a decrease of roughly 40% in hypoxia and CAIX expression, as measured by pimonidazole and CAIX immunohistochemical staining. This decrease was significant for both CAIX and pimonidazole, but was lower than expected. This difference is not as large as was described by McKenna et al. who measured a decrease of hypoxia of up to 90% in a FaDu xenograft model [17]. Atovaquone was administered with the same dosing-schedule as was used by McKenna et al. We treated mice for seven consecutive days with atovaquone, which should be long enough to account for the long half-life is the CAIX protein. Interestingly, the effect of hypoxia-modulating treatments was not equal in both tumor models. Especially, treatment with atovaquone did not achieve a reduction of hypoxia or CAIX expression in the SCCNij202 model, although both models express similar amounts of hypoxia and CAIX in the control tumors. This underlines the differential responses that can be present to atovaquone hypoxia modulation between different tumors.

Our hypoxic breathing treatment (48 h, 8% O_2_) did not increase hypoxia nor CAIX expression. Of note, mice that were exposed to hypoxic breathing, did not eat and drink properly and lost body weight due to dehydration. This may have resulted in altered pharmacokinetics and altered perfusion of the tumors, as demonstrated by significantly altered kidney and liver accumulation of the radiotracer and enhanced necrosis in the viable tumor tissue (Supl. Fig. 3). This effect on tumor necrosis has not been reported elsewhere. The increased tumor necrosis in this group hinders reliable immunohistochemical analysis and is likely the cause of the unexpected decrease in hypoxia and CAIX expression. Together, this makes the results from this treatment group difficult to interpret. We were not able to determine the necrotic fractions in the SCCNij202 line due to high amount of keratinization (Sup. Fig. 2).

Overall ex vivo tracer uptake in FaDu tumors showed a modest correlation with CAIX expression. However, despite the reduction of CAIX expression in the FaDU model, tumor tracer uptake did not decrease in the groups treated with atovaquone. High accuracy of quantitative CAIX assessment is warranted to enable detection of slight alterations in CAIX expression. Unfortunately, tumor accumulation of radiotracers is dependent on more variables than the amount of target antigen expressed in a tumor. As described by Xenaki et al. both tumor characteristics and tracer characteristics play a role in the amount of tracer accumulation in the tumor. Besides antigen availability, the main factors within a tumor are vessel density, vessel permeability, perfusion rate, perfusion volume and necrosis. As shown in Fig. 1, atovaquone treatment also influenced the vessel density of the FaDu model. With this influence on the tumor vasculature it is possible that atovaquone treatment also has influence on vascular permeability. Vascular permeability is known to be associated with an enhanced permeability and retention effect (EPR-effect) [18], [19]. Therefore, effects of atovaquone may have resulted in enhanced permeability and could be responsible for the higher ex vivo tracer uptake observed in our study, thereby masking a decrease in CAIX expression.

Our previous study on [^111^In]In-G250-F(ab’)_2_ demonstrated the capability to discriminate between low and high CAIX-expressing tumors, but also showed a significant level of non-specific uptake at 24 h p.i. The EPR effect has shown to be size-dependent and affects molecules larger than ~ 40 kDa, and thus affects F(ab’)_2_ which has a size of 110 kDa. As a result, the non-specific tracer uptake will hinder the accuracy of the CAIX quantification using these tracers particularly, when the vascular permeability changes due to therapeutic intervention. Even smaller radiotracers such as nanobody-based or affibody-based molecules are less affected by the EPR-effect [14], [20]. Future studies could investigate CAIX-targeting radiotracers based on nanobodies and test them in a similar hypoxia modifying experiment as presented here.

## Declaration of Competing Interest

The authors declare that they have no known competing financial interests or personal relationships that could have appeared to influence the work reported in this paper.
