# Supplementary material for: Imaging carbonic anhydrase IX as a method for monitoring hypoxia-related radioresistance in preclinical head and neck cancer models
Source: Phys Imaging Radiat Oncol. 2021 Aug 24;19:145–50. doi: 10.1016/j.phro.2021.08.004 (PMC8397885; doi:10.1016/j.phro.2021.08.004)
Supplement: Supplementary data 1 [file mmc1.docx]

**Supplementary figures**


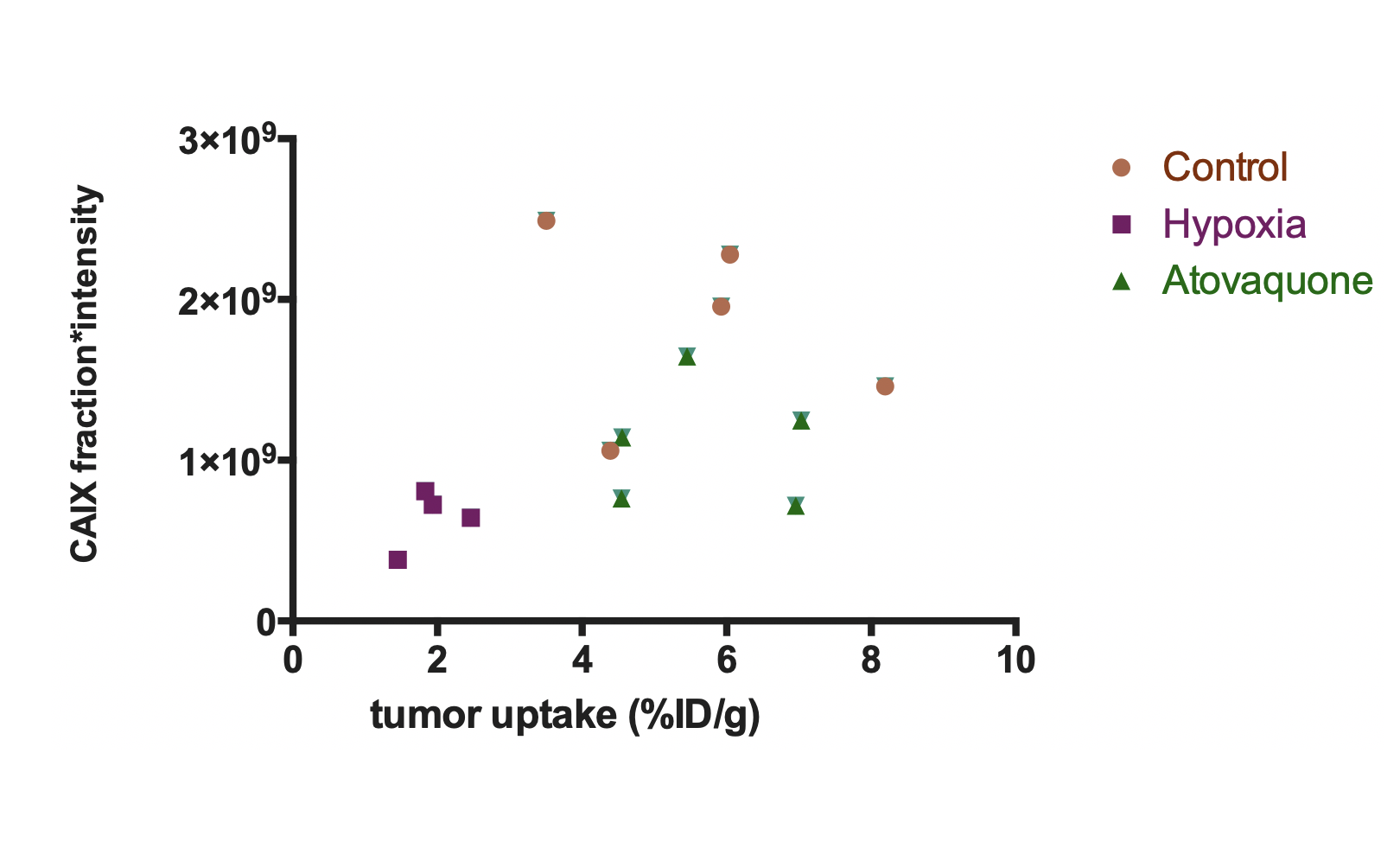


Sup. Figure 1. Correlation of ex vivo tumor tracer uptake (%ID/g) with CAIX fraction times signal intensity of all treatment groups of the FaDu tumor model. Pearson correlation coefficient r=0.49 (p = 0.08)


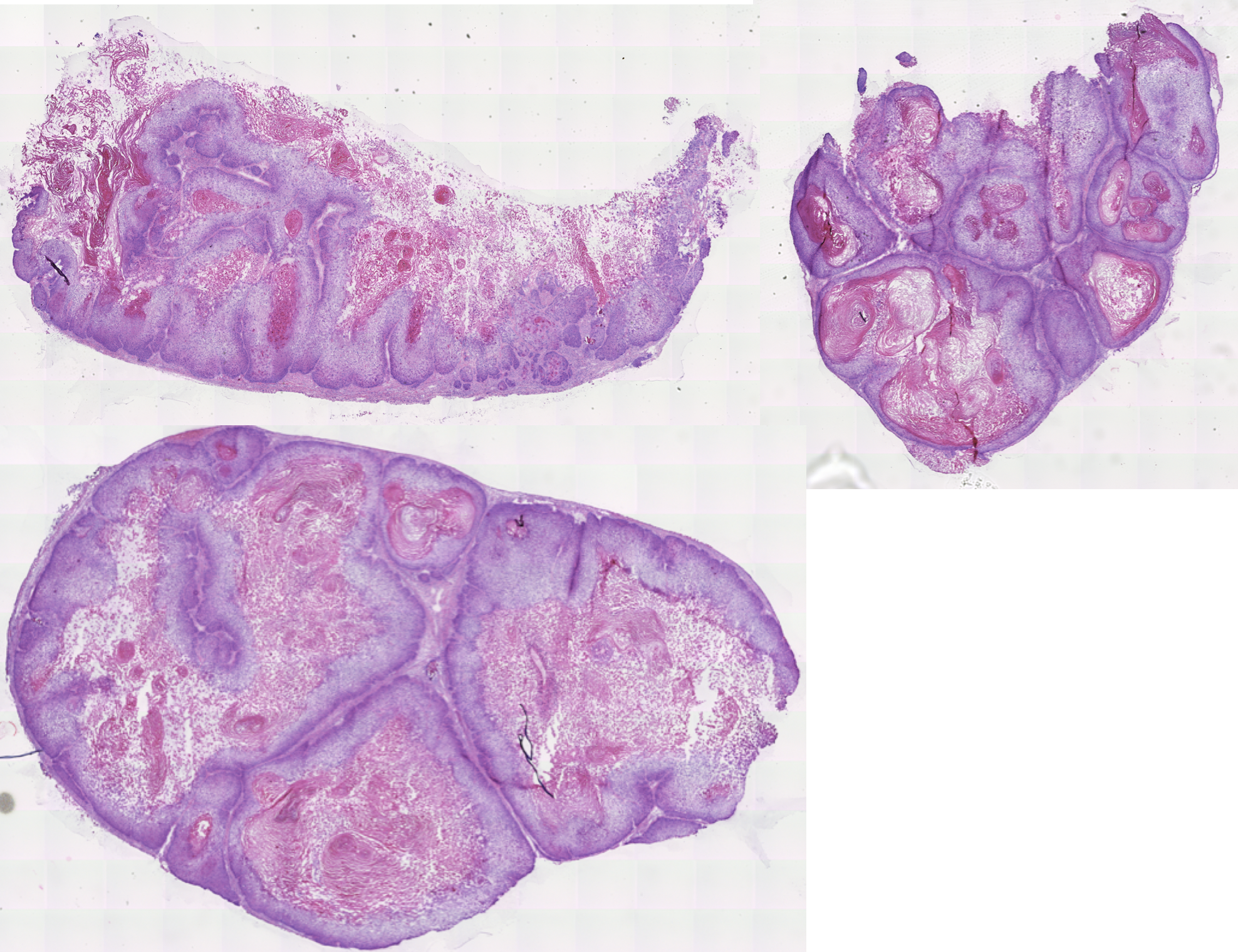


Sup. Figure 2. Haematoxylin and eosin staining of two SCCNij202 tumor sections. Section on the left is from the control group, the section on the right of a tumor treated with hypoxic breathing and the lower tumor section was treated with atovaquone.

Sup. Figure 3. Biodistribution profiles of all 3 treatment groups: Control, Hypoxic breathing and atovaquone (FaDu tumor model).
